# Spanish Ketogenic Mediterranean diet: a healthy cardiovascular diet for weight loss

**DOI:** 10.1186/1475-2891-7-30

**Published:** 2008-10-26

**Authors:** Joaquín Pérez-Guisado, Andrés Muñoz-Serrano, Ángeles Alonso-Moraga

**Affiliations:** 1 Department of Genetic, University of Córdoba, Campus of Rabanales, Córdoba, Spain

## Abstract

**Background:**

Ketogenic diets are an effective healthy way of losing weight since they promote a non-atherogenic lipid profile, lower blood pressure and decrease resistance to insulin with an improvement in blood levels of glucose and insulin. On the other hand, Mediterranean diet is well known to be one of the healthiest diets, being the basic ingredients of such diet the olive oil, red wine and vegetables. In Spain the fish is an important component of such diet. The objective of this study was to determine the dietary effects of a protein ketogenic diet rich in olive oil, salad, fish and red wine.

**Methods:**

A prospective study was carried out in 31 obese subjects (22 male and 19 female) with the inclusion criteria whose body mass index and age was 36.46 ± 2.22 and 38.48 ± 2.27, respectively. This Ketogenic diet was called "Spanish Ketogenic Mediterranean Diet" (SKMD) due to the incorporation of virgin olive oil as the principal source of fat (≥30 ml/day), moderate red wine intake (200–400 ml/day), green vegetables and salads as the main source of carbohydrates and fish as the main source of proteins. It was an unlimited calorie diet. Statistical differences between the parameters studied before and after the administration of the "Spanish Ketogenic Mediterranean diet" (week 0 and 12) were analyzed by paired Student's *t *test.

**Results:**

There was an extremely significant (p < 0.0001) reduction in body weight (108.62 kg→ 94.48 kg), body mass index (36.46 kg/m^2^→31.76 kg/m^2^), systolic blood pressure (125.71 mmHg→109.05 mmHg), diastolic blood pressure (84.52 mmHg→ 75.24 mmHg), total cholesterol (208.24 mg/dl→186.62 mg/dl), triacylglicerols (218.67 mg/dl→113.90 mg/dl) and glucose (109.81 mg/dl→ 93.33 mg/dl). There was a significant (p = 0.0167) reduction in LDLc (114.52 mg/dl→105.95 mg/dl) and an extremely significant increase in HDLc (50.10 mg/dl→54.57 mg/dl). The most affected parameter was the triacylglicerols (47.91% of reduction).

**Conclusion:**

The SKMD is safe, an effective way of losing weight, promoting non-atherogenic lipid profiles, lowering blood pressure and improving fasting blood glucose levels. Future research should include a larger sample size, a longer term use and a comparison with other ketogenic diets.

## Background

The international consensus is that carbohydrates are the basis of the food pyramid for a healthy diet and that the best way to lose weight is by cutting back on calories chiefly in the form of fat. It is generally believed that ketogenic diets may lead to the development of several diseases. However, many studies have found that ketogenic diets are healthier since they help to preserve muscle mass, reduce appetite, diminish metabolic efficiency, induce metabolic activation of thermogenesis, favor increased fat loss, promote a non-atherogenic lipid profile, lower blood pressure and decrease resistance to insulin with an improvement in blood levels of glucose and insulin [1]. Contrary to past opinions, high carbohydrate diets may be associated with: low levels of high-density lipoprotein cholesterol (HDLc), high levels of triacylglycerols (TG), low-density lipoprotein cholesterol (LDLc) and total cholesterol [2], type 2 diabetes mellitus [3], metabolic syndrome, essential hypertension [4] and cancer [5].

Mediterranean diet has evident health benefits. Such diet is associated with a longer life span [6,7] and lower rates of coronary heart disease, certain cancers [8], hypercholesterolemia, hypertension, diabetes and obesity [9]. It is difficult to define which are the healthiest constituents of the Mediterranean diet, since it is a very varied diet that can change among the Mediterranean countries. For example, in Spain the fish is an important component [10,11] as well as the olive oil, red wine and vegetables, that are 3 essential components of such diet in all the countries. The healthy properties of the incorporation of olive oil, red wine and fish consumption to a ketogenic diet could be explained by the 3 following sections. Regarding the healthy properties of vegetables it is well known that they are high in water, phytonutrients, antioxidants and provide a good source of fiber.

The objective of the present study was to determine the dietary effects of the "Spanish Ketogenic Mediterranean Diet" (SKMD). Such diet was a protein ketogenic diet under free-living conditions with 4 important healthy components of the Mediterranean diet in Spain: olive oil, salad, fish and red wine. Therefore, the present study was carried out to demonstrate the changes in body weight, blood pressure, lipid profile and glucose that might occur after the administration of SKMD throughout the period of study (12 weeks), in healthy obese subjects.

### Olive oil

Olive oil, is considered the pillar of the Mediterranean diet, since it improves the major risk factors for cardiovascular disease, such as the lipoprotein profile, blood pressure, glucose metabolism and antithrombotic profile. Endothelial function, inflammation and oxidative stress are also positively modulated. Some of these effects are attributed beside the monounsaturated fatty acids (MUFA) to the minor components of virgin olive oil [12]. Hydrocarbons, polyphenols, tocopherols, sterols, triterpenoids and other components, despite their low concentration, non-fatty acid constituents may be of importance because studies comparing monounsaturated dietary oils have reported different effects on cardiovascular disease. Most of these compounds have demonstrated antioxidant, anti-inflammatory and hypolipidemic properties [13]. Moreover, MUFA-rich diet prevents central fat redistribution and the postprandial decrease in peripheral adiponectin gene expression and insulin resistance induced by a carbohydrate-rich diet in insulin-resistant subjects [14].

### Red wine

The combination of ethanol and phenolic compounds in red wine is thought to be responsible for the apparent protective cardiovascular effect [15], showing olive oil and red wine antioxidant polyphenols antiatherogenic properties [16]. Moreover, combined consumption of wine and olive oil provided beneficial postprandial effects on haemodynamics [17].

### Fish

Two long-chain Omega-3 polyunsaturated fatty acids (n-3 PUFA), eicosapentaenoic acid (EPA) and docosahexaenoic acid (DHA), are the active constituents of the fish. Low rates of death from coronary heart disease has been report among individuals with very high consumption of fish, although these people should limit intake of species highest in mercury levels. Larger, longer-living predators (swordfish, shark) have higher tissue concentrations, while smaller or shorter-lived species (anchovy, shellfish, salmon, sardine) have very low concentrations [18].

High omega-3 consumption increases insulin sensitivity and reduces inflammatory markers [19] and Piers et al. have hypothesized that unsaturated fats (MUFA and/or PUFA), rather than saturated fat (SFA), are more effective in stimulating peroxisome proliferator-activated receptor-α leading to fat oxidation, with SFA being much more readily diverted to fat storage [20].

## Methods

### Subjects

A prospective study was carried out at a General Medicine Consultation (Córdoba, Spain) in 40 overweight subjects (22 male and 19 female) whose body mass index and age was 36.46 ± 2.22 and 38.48 ± 2.27, respectively. Subjects were selected with the cooperation of a database medical weight loss clinic. Inclusion criteria were: a diet based on carbohydrate foods (> 50% of dairy energy intake), achievement of desired weight loss, normal liver and renal function, not to have antecedents of gout or high uric acid, not to have exercise, alcoholic and smoking habits, not to be pregnant or lactating, IMC ≥ 30, age ≥ 18 years and ≤ 65 years and not to be under medication. Since obesity increases the risk for alterations in hepatocyte function that lead to accumulation of lipid in hepatocytes and hepatomegaly (Non-alcoholic Fatty Liver Disease), we consider higher liver transaminase levels as a variant of normality in such obese patients (hepatic transaminases ≤ twice normal values → GOT and GPT ≤ 80 mU/ml). Chronic hepatitis B or C was ruled out in such patients by negative serologies. We determined normal renal function as measured by plasma urea nitrogen and plasma creatinine: creatinine ≤ 1.3 mg/dl and urea ≤ 40 mg/dl. Subjects with the inclusion criteria were selected for eligibility by phone and 40 eligible subjects were invited to attend an orientation session during the week prior to the study. Patients measured their body's ketosis state every morning by ketone strips. During the study, the participants were phoned by the same person weekly, in order to assure the correct realization of the protocol and the ketosis state. If the subjects failed to maintain adequate compliance with the clinical trial protocol they would be dropped out the study.

Subjects received no monetary compensation for their participation and provided voluntary written consent form before initiating the diet.

The Ethics and Clinical Investigation Committee of the "Spanish Medical Association of the Proteinic Diet" approved the study protocol, informed consent form and subject informational materials. Patient anonymity was preserved.

### Diet

This protein ketogenic diet was called "Spanish Ketogenic Mediterranean Diet" (SKMD) due to the incorporation of virgin olive oil as the principal source of fat, moderate red wine intake, green vegetables and salads as the main source of carbohydrates and fish as the main source of proteins. It was an unlimited calorie diet, nevertheless subjects were encouraged to consume per day: a maximum of 30 g of carbohydrates in the form of green vegetables and salad, a minimum of 30 ml of virgin olive oil, 200–400 ml of red wine and no limit of the protein block.

Participants were permitted 3 portions (200 g/portion) of vegetables daily: 2 portions of salad vegetables (such as alfalfa sprouts, lettuce, escarole, endive, mushrooms, radicchio, radishes, parsley, peppers, chicory, spinach, cucumber, chard and celery), and 1 portion of low-carbohydrate vegetables (such as broccoli, cauliflower, cabbage, artichoke, eggplant, squash, tomato and onion). 3 portions of salad vegetables were allowed only if the portion of low-carbohydrate vegetables were not consumed. Salad dressing allowed were: garlic, olive oil, vinegar, lemon juice, salt, herbs and spices.

The minimum 30 ml of olive oil were distributed unless in 10 ml per principal meal (breakfast, lunch and dinner). Red wine (200–400 ml a day) was distributed in 100–200 ml per lunch and dinner. The protein block was divided in "fish block" and "no fish block". The "fish block" included all the types of fish except larger, longer-living predators (swordfish and shark). The "no fish block" included meat, fowl, eggs, shellfish and cheese. Both protein blocks were not mixed in the same day and were consumed individually during its day on the condition that at least 4 days of the week were for the "fish block".

Trans fats (margarines and their derivatives) and processed meats with added sugar were not allowed.

No more than two cups of coffee or tea and at least 3 litres of water were intake each day. Infusions and artificial sweeteners were allowed (saccharin, cyclamate, acesulfame, aspartame and sucralose).

### Supplements

Micronutrients (vitamins and minerals) were given daily to each subject in the form of 2 tablets of a poly-vitamin-mineral supplement and one tablet of calcium carbonate 1500 mg. Each tablet of the poly-vitamin-mineral supplement contained: vitamin A 680 mcg, Beta-carotene 720 mcg, vitamin D 5 mcg, vitamin E 10 mg, vitamin C 60 mg, vitamin B_1 _1.4 mg, vitamin B_2 _1.6 mg, vitamin B_6 _2 mg, folic acid 200 mcg, vitamin B_12 _1 mcg, niacin 18 mg, biotin 150 mcg, pantothenic acid 6 mg, vitamin K 30 mcg, calcium 120 mg, potassium 40 mg, phosphorus 126.3 mcg, iron 8 mg, magnesium 45 mg, cupper 0.9 mg, zinc 8 mg, manganesum 1.8 mg, iodine 75 mcg, molibden 45 mcg, boron 70 mcg, chlorine 21 mg, chromium 25 mcg, molybdenum 45 mcg, nickel 5 mcg, selenium 55 mcg, silicon 3 mg, sin 10 mcg, vanadium 10 mcg.

### Measurements

Subjects were weighed and systolic/diastolic blood pressure was measurement at weeks 0, 4, 8 and 12, at the same time (that depends on the subject) and using always the same digital scale ("Seca 703") and mercurial sphygmomanometer ("Labtron Model 03-225").

Fasting venous blood samples were collected at weeks 0 and 12 for total cholesterol, HDLc, LDLc, triacylglycerol and glucose. Venous blood samples for glucose, lipid and lipoprotein analysis were collected into EDTA-containing (1 g/l) tubes from all subjects after a 12 h overnight fast at the beginning of the study and at the end of each dietary period. Plasma was obtained by low-speed centrifugation for 15 min at 4°C within 1 h of venepuncture. Plasma cholesterol and TAG levels were determined by enzymatic techniques. HDL-cholesterol was determined after precipitation with fosfowolframic acid LDL-cholesterol concentration was calculated using the Friedewald formula. Plasma glucose was measured by the glucose oxidase method. To reduce interassay variation, plasma was stored at -80°C and analysed at the end of the study.

### Statistical analysis

Statistical differences between the parameters before and after the administration of the SKMD (week 0 and 12) were analyzed by paired Student's *t *test with SPSS 12.0 (SPSS Inc., Chicago, IL, USA) and are expressed as mean ± standard error of the mean (SEM). The parameters studied were: weight, body mass index (BMI), systolic blood pressure (SBP), diastolic blood pressure (DBP), total cholesterol, HDLc, LDLc, triacylglycerol and glucose. Before the Student's *t *test, Kolmogorov-Smirnov and Shapiro-Wilk tests were used for testing normality and the assumption of homoscedasticity was determined with the F-Snedecor test.

## Results

### Subject attrition

Of the 40 persons who started the study, data collected from 31 subjects were used in the final analysis. Data were not use from 9 subjects: 3 subjects were withdrawn for failure to maintain adequate compliance with the clinical trial protocol; 4 subjects were lost to follow-up; 1 subject withdrew because he said the diet was too expensive; 1 subject was withdrawn due to suffer a polytraumatism car accident.

### Parameters analyzed

Normal distribution and the assumption of homoscedasticity were verified. As there were no significant differences in male and female subjects in all the parameters examined (p > 0.05), the data of males and females in each group are pooled and presented together. The changes in all the parameters studied are shown in Table 1.

**Table 1:** Changes in the level of various parameters before and after the SKMD

**Parameters**	Week 0*	Week 12*	**% of Change**	**P**
Weight (kg)	108.62 ± 3.18	94.48 ± 2.83	13.02	<0.0001

BMI (kg/m^2^)	36.46 ± 0.84	31.76 ± 0.74	12.89	<0.0001

Total Cholesterol (mg/dl)	208.24 ± 5.86	186.62 ± 5.80	10.38	<0.0001

LDLc (mg/dl)	114.52 ± 6.17	105.95 ± 7.67	7.48	0.0167

HDLc (mg/dl)	50.10 ± 1.69	54.57 ± 1.50	8.19	<0.0001

Triacylglicerols (mg/dl)	218.67 ± 9.55	113.90 ± 5.20	47.91	<0.0001

Glycemia (mg/dl)	109.81 ± 2.22	93.33 ± 1.83	15.01	<0.0001

SBP (mm Hg)	125.71 ± 5.19	109.05 ± 4.41	13.25	<0.0001

DBP (mm Hg)	84.52 ± 2.76	75.24 ± 2.35	10.98	<0.0001

*Data are expressed as mean ± standard error of the mean (SEM).

There was an extremely significant (p < 0.0001) reduction in body weight (108.62 kg→ 94.48 kg), BMI (36.46 kg/m^2^→31.76 kg/m^2^), SBP (125.71 mmHg→109.05 mmHg), DBP (84.52 mmHg→ 75.24 mmHg), total cholesterol (208.24 mg/dl→186.62 mg/dl) and glucose (109.81 mg/dl→ 93.33 mg/dl). There was a significant (p = 0.0167) reduction in LDLc (114.52 mg/dl→105.95 mg/dl) and an extremely significant increase in HDLc (50.10 mg/dl→54.57 mg/dl).

## Discussion

### Weight loss

It is thought that consumption of a high-fat-protein diet will be accompanied by a higher weight gain. On the contrary, our results confirm that the SKMD is an effective therapy for obesity without caloric restriction. This might be due to the fact that there is a synergic effect between the high protein ketogenic nature of the diet and its richness in MUFA and PUFA. We don't have data about the percentage of body fat and lean body mass lost. Nevertheless we think that there was a more selective fat loss because we didn't observe the flaccidity physical aspect that we have observed before with hypocaloric diets, and subjects had a physical aspect similar to a liposuction, since fat was removed from many different fat specific deposit areas, including the abdomen, thighs, hips, buttocks, waist, neck and upper arms. Our hypothesis is founded in the following statements:

1. Many studies have confirmed that the ketogenic diet is an effective therapy for obesity [1,21-26]. In addition to the fact that an equal number of calories are ingested, ketogenic diets are more effective for achieving fat loss than the conventional high-carbohydrate/low-fat diets [1,26]. Low-carbohydrate diets have even proved to be more effective than conventional diets for more selective fat loss and conserving muscle mass [1,24-26], moreover, several longer term studies have noted improvements in body composition on a higher protein pattern despite similar weight loss [27].

2. MUFA-rich diet prevents central fat redistribution [14].

3. High unsaturated fat diet is more effective to preserve lean mass than a low fat diet or a low carbohydrate diet [23]. Moreover, the PUFA from the fish, DHA and EPA exhibit "anti-obesity" effect as well as improving insulin sensitivity [28].

In connection with the moderate red wine consumption of the SKMD, we agree with the statement that moderate red wine consumption (450 ml) is not associated with differences in body weight [29], so this consumption would not affect to the weight loss.

Further trials are required to examine the potential role of the SKMD for the selective fat loss and its protective effect against muscle protein catabolism.

### Glycemic control

During the SKMD the fasting glycemia improved significantly. These findings could be explained by the following points:

1. A low carbohydrate diet reduces fasting glucose levels, even independently of the weight loss [30,31].

2. MUFA-rich diet prevents insulin resistance induced by a carbohydrate-rich diet in insulin-resistant subjects [14].

3. DHA-EPA also improve insulin sensitivity [28].

Our data are not enough to state with precision if the SKMD is the same or better than a conventional ketogenic diet to improve glycemic control due to its higher content in MUFA and DHA-EPA.

We think that the moderate prandial red wine consumption of the SKMD did not have effect (beneficial or adverse) on the glycemic control, since Gin et al. reported that moderate prandial wine consumption has no adverse effect on the glycemic control of diabetic patients, thus it appears unnecessary to proscribe the consumption of red wine in moderation with meals to diabetic patients [32].

### Effects of the "Spanish Ketogenic Mediterranean diet" on cardiovascular parameters

The data presented in this study showed that the SKMD significantly decreases the total cholesterol, LDLc, triacylglycerols, SBP, DBP and increases the level of HDLc. This healthy cardiovascular profile is probably due to the favorable interaction of the weight loss and the components of the SKMD: high protein ketogenic diet-virgin olive oil-fish oil-red wine-salad. Our arguments are founded in the following findings:

1. Ketogenic diets improve all aspects of atherogenic dyslipidemia, decreasing fasting and postprandial triglyceride levels and increasing HDLc and LDLc particle size [1,33]. When the ketogenic diet is higher in protein than fat, the level of LDLc also decreases [33-35].

2. Ingestion of virgin olive oil increases HDLc levels [36] and decreases LDLc levels [37-39] and blood pressure [38,39].

3. Omega-3 PUFA reduce plasma triacylglycerol concentrations [40,41].

4. Chronic moderate consumption of red wine (400 ml/day) significantly reduces fasting LDLc and increases HDLc in hypercholesterolaemic patients [42].

5. Low carbohydrate/high-protein diets are more effective than high-carbohydrate diets for decreasing blood pressure, both diastolic and systolic [43].

6. The salad consumption is inversely associated with diastolic blood pressure [44].

### Explanations and Suggestions

We recognize several limitations of our study that may have influenced the study findings:

1. The sample of the study is small (31 subjects).

2. This is not a random population study, since subjects were selected for eligibility and their eligibility was related with their compliance to the diet.

3. Weight loss may be related with improvement in all parameters that are studied.

4. We didn't take into consideration calories intake before and after the 12 weeks. Although it is known that an equal number of calories are ingested, ketogenic diets are more effective for achieving fat loss than the conventional high-carbohydrate/low-fat diets [1,26], we don't know if our patients intake less food and calories, and if it is the case, this would be correlated with weigh reduction and better cardiovascular parameters.

5. Although the effect of vitamins is not clear, especially in short interventions, their possible contribution to better cardiovascular parameters should be possible.

6. Our study has no control groups to consider the interaction between the components of the SKMD. There is no way to say if the healthy results are due to the ketogenic nature of the diet, the virgin olive oil, the red wine, the higher fish intake, the higher salad intake or a synergic effect between these components.

All these limitations should be known and accordingly considered by further trials.

## Conclusion

The SKMD is safe, an effective way of losing weight, promoting non-atherogenic lipid profiles, lowering blood pressure and improving fasting blood glucose levels. Future research should include a larger sample size, a longer term use and a comparison with other ketogenic diets.

## Abbreviations

EPA: eicosapentaenoic acid; DHA: docosahexaenoic acid; HDLc: high-density lipoprotein cholesterol; LDLc: low-density lipoprotein cholesterol; MUFA: monounsaturated fatty acids; PUFA: polyunsaturated fatty acids; SFA: saturated fat; SKMD: Spanish Ketogenic Mediterranean Diet; TG: triacylglycerols.

## Competing interests

The authors declare that they have no competing interests.

## Authors' contributions

JPG was the principal researcher and was responsible for study design, acquisition of data, analysis and interpretation of data and preparation of manuscript. AMS was responsible for analysis and interpretation of data. AAM was responsible for study design, analysis and interpretation of data.
